# Cellular Senescence and the SASP in HFpEF: Pathogenic Mechanisms and Therapeutic Targeting

**DOI:** 10.3390/ijms27125278

**Published:** 2026-06-10

**Authors:** Qiu-Cheng Zhu, Qiong Zheng, Hao Zhang, Yan Tu, Ping Wang, Xiang-Jie Liu

**Affiliations:** 1 Li-Yuan Hospital, Tongji Medical College, Huazhong University of Science and Technology, Wuhan 430077, China; 18337328327@163.com (Q.-C.Z.); zq13026382296@163.com (Q.Z.); 13971129375@163.com (H.Z.); tuyan7901@126.com (Y.T.); 2 Laboratory of Metabolic Abnormalities and Vascular Aging, Huazhong University of Science and Technology, Wuhan 430077, China

**Keywords:** HFpEF, SASP, cellular senescence, senolytics, senomorphics

## Abstract

Heart failure with preserved ejection fraction (HFpEF) represents a complex syndrome strongly associated with aging, characterized by diastolic dysfunction, myocardial stiffness, and chronic low-grade inflammation. Cellular senescence and the ensuing senescence-associated secretory phenotype (SASP) significantly contribute to the pathogenesis and progression of HFpEF. This review examines the biological properties of SASP and its mechanistic roles in driving myocardial fibrosis, microvascular dysfunction, and cardiomyocyte injury. We synthesize evidence from preclinical and clinical studies demonstrating how SASP factors orchestrate HFpEF pathophysiology. The therapeutic potential of targeting SASP pathways is critically evaluated, including senolytic agents that eliminate senescent cells and senomorphic compounds that inhibit SASP factor secretion. Finally, we identify key translational barriers, such as limited tissue specificity in senolytic delivery and inadequate SASP biomarkers for treatment monitoring, while outlining future research directions to advance novel therapeutic development for this increasingly prevalent condition.

## 1. Introduction

Heart failure has recently been classified into four phenotypes based on left ventricular ejection fraction (LVEF) in patients with symptomatic HF: HF with reduced EF (HFrEF, LVEF ≤ 40%), HF with mildly reduced EF (HFmrEF, LVEF 41–49%), and HF with preserved EF (HFpEF, LVEF ≥ 50%). Additionally, HF with improved EF (HFimpEF) is defined as symptomatic HF with a baseline LVEF ≤ 40% that subsequently increases by ≥10 percentage points and to a value >40% [1]. HFpEF accounts for more than 50% of all heart failure cases. Its prevalence increases markedly with age [2,3], posing a major public health burden worldwide. HFpEF exhibits a more complex, aging-associated pathological mechanism characterized by systemic inflammation and multi-organ dysfunction. Cellular senescence has garnered increasing attention in cardiovascular aging research. Senescent cells accumulate in aging tissues and represent a state of permanent cell cycle arrest in response to damage. While this process can suppress tumor growth and exert beneficial effects, it may also impair tissue regeneration and promote inflammation and disease [4]. Notably, the accumulation of senescent cells and their secretion of the SASP within the cardiovascular system may form a critical link between aging and HFpEF pathology [5]. SASP promotes a dysfunctional microenvironment and myocardial fibrosis through the release of pro-inflammatory factors, chemokines, and extracellular matrix-remodeling proteins [6], which is considered a central driver of HFpEF. This contrasts with HFrEF, which typically results from acute cardiomyocyte loss and ischemic injury. This distinction is critically reflected in the role of cellular senescence and the SASP, which are now recognized as central amplifiers of myocardial stiffness, microvascular impairment, and metabolic dysregulation in HFpEF, with features less dominant in classical HFrEF pathogenesis. The limited treatment options for HFpEF stem from both an incomplete understanding of the complex, multifactorial pathophysiology, in which SASP is a key but not exclusive driver, and a lack of robust evidence from large, randomized trials targeting these specific pathways. Patients typically exhibit structural abnormalities such as ventricular wall thickening, myocardial fibrosis, and left ventricular hypertrophy [7,8], which often coincide with metabolic disorders including obesity, diabetes, and hypertension [9,10,11]. However, the specific mechanisms through which these comorbidities lead to cardiac impairment remain unclear. Current interventions targeting SASP show therapeutic potential. Further research should focus on clarifying SASP-related mechanisms, developing targeted strategies against inflammation, and translating these findings into clinical applications. Overcoming these challenges is essential to providing more effective treatments for HFpEF.

This review aims to (1) outline the core signaling pathways regulating SASP in the heart, (2) critically evaluate the evidence linking SASP to the three pillars of HFpEF pathophysiology, and (3) summarize the state of therapeutic translation. It is important to note that while a definitive causal link between cellular senescence and HFpEF in humans remains to be fully established, this review synthesizes the compelling associative and preclinical interventional evidence that positions the SASP as a plausible and potent driver of HFpEF pathophysiology, thereby justifying its investigation as a therapeutic target.

## 2. Biology of Cellular Senescence and SASP

### 2.1. Cellular Senescence: Cardiovascular Inducers

Cellular senescence is defined as stable, long-term cell cycle arrest rather than absolutely irreversible growth arrest under all biological contexts [12,13]. Its mechanisms primarily involve the classic telomere-dependent pathway, wherein telomeres shorten with each cell division until reaching a critical length that triggers senescence, as well as telomere-independent pathways, such as oxidative damage, mitochondrial dysfunction, and chronic inflammation, which can induce senescence even when telomeres remain intact [12]. Based on the triggers, cellular senescence can be classified into four major types: replicative senescence (RS) [14], oncogene-induced senescence (OIS) [15,16,17], therapy-induced senescence (TIS) [16], and mitochondrial dysfunction-associated senescence (MiDAS) [18]. Despite their different origins, all types share hallmark features, including cell cycle arrest, morphological changes, telomere shortening, senescence-associated heterochromatin foci (SAHF) formation, the SASP, and specific molecular alterations [19]. Furthermore, senescence cells exhibit functional and phenotypic heterogeneity depending on the initial stimulus.

In the cardiovascular system, senescence can be induced by multiple factors: oxidative stress increases reactive oxygen species (ROS), causing direct damage to DNA and proteins and reducing antioxidant enzyme activity, which activates the p53-p21 pathway leading to cell cycle arrest [20,21]; hemodynamic stress such as hypertension-induced endothelial mechanical stretching and turbulent shear stress promote telomere shortening and inflammatory activation [22,23]; metabolic insults, including hyperglycemia-induced accumulation of advanced glycation end products (AGEs) that activate RAGE receptors and cause mitochondrial damage and DNA breaks, hyperlipidemia leading to ox-LDL uptake by macrophages to form foam cells and release Interleukin-1β/tumor necrosis factor-α (IL-1β/TNF-α) promoting vascular cell senescence, and insulin resistance disrupting the Phosphoinositide 3-Kinase (PI3K)/Akt signaling pathway and impairing cellular repair, also contribute to senescence [24].

Genotoxic stressors and clinical treatments, such as radiotherapy and chemotherapeutic agents (e.g., doxorubicin) [25], directly damage cardiomyocyte DNA and accelerate telomere attrition, thereby reducing the replicative potential [26]. Additionally, a chronic inflammatory milieu containing cytokines such as TNF-α and interleukin-6(IL-6) promotes senescence through the activation of Nuclear Factor Kappa-Light-Chain-Enhancer of Activated B Cells (NF-κB), and activated T cells secreting Interferon-gamma (IFN-γ) can induce SASP, forming a vicious cycle [27].

In summary, cellular senescence is a complex biological process regulated by diverse intrinsic and extrinsic factors. Within the cardiovascular system, senescence not only impairs normal cardiac and vascular function but also contributes critically to the pathogenesis of multiple cardiovascular diseases. In patients with HFpEF, cardiomyocytes and cardiac fibroblasts are continuously exposed to sublethal injuries, potentially placing them in a transitional state of “dynamic senescence.” In this state, cells exhibit G1 phase arrest, increased senescence-associated β-galactosidase (SA-β-Gal) activity, and SASP, yet their growth arrest is not absolutely irreversible. Indeed, certain interventions, such as targeted clearance of senescent cells (senolytics) or removal of underlying pathogenic stressors (e.g., weight loss, blood pressure control), may restore function in some cells. Understanding the triggers and mechanisms driving cellular senescence thus holds significant clinical value for developing novel therapeutic approaches against cardiovascular disorders.

### 2.2. Composition and Dynamics of the SASP

The SASP represents a characteristic secretory profile of senescent cells that functions as a molecular communication toolkit [28]. Its most well-characterized components are proteins [17], including inflammatory cytokines [29], such as interleukins [30], chemokines [31], growth factors, and extracellular matrix proteases [32]. In addition, bioactive lipids [33], extracellular vesicles (EV) [33,34], and non-coding RNAs contribute to the paracrine signaling effects of senescent cells [35].

This granular understanding is complemented by the study of EV (exosomes). These 30–150 nm vesicles, formed via late endosome involution and carrying proteins, RNAs, and lipids, are key mediators of non-cell-autonomous signaling [34,36]. In senescence, exosomes propagate aging signals systemically, and their SASP factor cargo serves as a promising reservoir for biomarker discovery in HFpEF, reflecting underlying immune-metabolic dysregulation [37]. These technologies collectively reveal that aging signals extend far beyond the heart. Notably, circulating SASP factors and exosomes facilitate critical crosstalk with distant tissues, such as adipose tissue, thereby exacerbating systemic metabolic dysfunction and myocardial fibrosis [38]. This multi-organ perspective is vital for understanding HFpEF as a systemic disorder.

A limited amount of SASP factors may also be produced by non-senescent cells, including immune cells. These molecules work in concert to establish a sophisticated regulatory network: pro-inflammatory cytokines mediate inflammatory responses and modulate cell signaling [17], chemokines recruit immune cells to orchestrate local inflammation [39], Matrix Metalloproteinases (MMPs) remodel the extracellular matrix to maintain tissue structure, and growth factors regulate processes such as proliferation, differentiation, and fibrosis [40,41]. Notably, SASP function exhibits spatiotemporal heterogeneity; it initially plays a protective role following tissue damage by promoting repair, such as MMP/Transforming growth factor-β(TGF-β)-driven wound healing, but transitions toward a pathological role with advancing age [42]. The sustained release of pro-inflammatory factors, including IL-6 and TNF-α, fosters chronic inflammation and thereby accelerates age-related pathologies such as cardiovascular disease [43].

In HFpEF, dynamic changes in SASP factors, for instance, IL-8-mediated recruitment of inflammatory cells into microvessels and TGF-β-driven cardiac fibrosis, act through pathways involving inflammation–metabolism imbalance and tissue remodeling [44], serving as a central mechanism impairing myocardial function. Thus, we summarize the major protein components of SASP and their pathological roles in HFpEF (Table 1).

## 3. Aging as a Central Driver of HFpEF Pathogenesis

### 3.1. Cellular Senescence and SASP: Linking Aging to HFpEF Heterogeneity

HFpEF is a clinically heterogeneous syndrome, and several phenotype classifications have been proposed to capture its diverse clinical presentations. Based on the predominant underlying etiologies and associated comorbidities, HFpEF can be broadly categorized into at least three distinct but often overlapping phenotypes: (1) a metabolic phenotype driven by obesity, diabetes, and insulin resistance; (2) a hypertension-driven phenotype characterized by long-standing pressure overload and Renin–Angiotensin–Aldosterone System (RAAS) activation; and (3) an age-related phenotype primarily associated with the natural accumulation of senescent cells, often presenting with multi-organ decline and primary diastolic dysfunction [50]. These phenotypes share common downstream pathophysiological mechanisms, yet each exhibits distinct upstream drivers, which have important implications for targeted therapies. In the following sections, we discuss how cellular senescence and SASP manifest across these phenotypic classes. HFpEF is fundamentally an age-associated disorder, and its clinical expression is heterogeneous, encompassing distinct phenotypes such as those driven by metabolic dysfunction or vascular stiffness [50]. The incidence rises exponentially beyond the sixth decade of life. Epidemiological analyses indicate that more than 80% of HFpEF cases occur in individuals aged over 65 years, underscoring aging as the foremost risk factor for this syndrome [51]. Beyond mere chronological association, aging confers a distinct biological milieu characterized by chronic low-grade inflammation (“inflammaging”), progressive cellular senescence, and gradual decline in tissue resilience [52]. These processes converge to promote diastolic dysfunction, myocardial stiffening, and microvascular impairment-the cardinal pathophysiological hallmarks of HFpEF. Critically, the accumulation of senescent cells within cardiac and vascular tissues acts as a key mechanism linking aging to HFpEF. These cells secrete a robust SASP, comprising pro-inflammatory cytokines (IL-6, TNF-α, IL-1β), chemokines, and matrix-remodeling factors, which collectively sustain a local inflammatory microenvironment, drive fibrotic remodeling, and compromise endothelial function. Furthermore, aging intersects with and amplifies the impact of common HFpEF comorbidities-such as hypertension, diabetes, and obesity-each of which independently accelerates cellular senescence and amplifies SASP production [9,10,11].

HFpEF constitutes a clinically heterogeneous syndrome driven by diverse etiologies, with cellular senescence serving as a pivotal downstream mechanism. Aging actively establishes a pro-senescent framework that underlies disease onset and progression. This mechanism manifests distinctly across primary phenotypes. In the metabolic phenotype, visceral obesity and insulin resistance promote senescence primarily in adipose tissue macrophages and cardiac endothelial cells through mitochondrial dysfunction, while cardiomyocytes may also undergo senescence under chronic metabolic stress [53,54]. In the hypertension-driven phenotype, sustained hemodynamic stress and RAAS activation drive senescence in vascular smooth muscle cells and cardiac fibroblasts, contributing to arterial stiffness and myocardial fibrosis. Endothelial cells are also affected, leading to microvascular rarefaction [50]. The advanced age-related phenotype is primarily characterized by the natural accumulation of senescent cells, with cardiac fibroblasts, endothelial cells, and cardiomyocytes all showing increased senescence markers with advancing age [55]. Across all phenotypes, aging fundamentally amplifies these specific pathological pathways, with senescence providing a critical mechanistic link between varied etiologies and the unified clinical presentation of HFpEF [50]. In all cases, aging serves as the foundational amplifier of these phenotype-specific pathways. Thus, aging does not merely coincide with HFpEF but actively primes the myocardium for SASP-mediated damage, establishing a mechanistic framework through which senescence contributes to the onset, progression, and phenotypic severity of HFpEF.

A critical conceptual challenge in linking cellular senescence to HFpEF pathophysiology is the distinction between causation and correlation. While substantial evidence supports the notion that senescence and SASP drive myocardial fibrosis, microvascular dysfunction, and diastolic dysfunction, the inverse relationship is equally plausible: chronic diastolic dysfunction, persistent microvascular impairment, and the resultant biomechanical stress may themselves promote local inflammation and further accelerate cellular senescence [5]. Indeed, the pathophysiological changes in HFpEF are unlikely to follow a simple linear sequence; rather, they are governed by a complex regulatory network characterized by feedforward and feedback loops [12]. For example, SASP-induced microvascular dysfunction impairs oxygen delivery, leading to metabolic stress and additional mitochondrial DNA damage, which in turn activates the cGAS-STING pathway to perpetuate SASP secretion. This reciprocal amplification complicates the identification of upstream versus downstream events. From a therapeutic perspective, this distinction is crucial: targeting a downstream effector (e.g., a single SASP cytokine) may provide symptomatic relief, whereas eliminating upstream senescent cells (e.g., with senolytics) could theoretically break the vicious cycle at its source. We have endeavored throughout this review to distinguish established causal evidence (derived from genetic or pharmacological interventions in preclinical models) from associative findings, and we acknowledge that definitive proof of causation in human HFpEF remains an ongoing challenge [2].

### 3.2. Coordinated Signaling Pathways Driving HFpEF Pathogenesis

**Figure 1:**
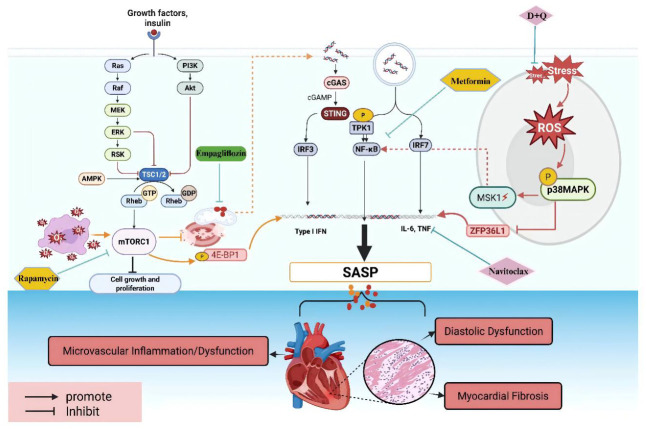
Core Regulatory Mechanisms and Therapeutic Targets in HFpEF. Solid arrows represent direct activation or promotion; dashed arrows indicate indirect or multi-step regulation; T-bars represent inhibition.

The pathogenesis of HFpEF involves a core regulatory network comprising the cyclic GMP–AMP synthase, stimulator of interferon genes (cGAS-STING) pathway [56,57], NF-κB [27,58], p38 mitogen-activated protein kinase (p38 MAPK) [59], and mechanistic target of rapamycin complex 1 (mTORC1) [60]. When cardiomyocytes experience DNA damage or mitochondrial DNA (mtDNA) release, cGAS-STING activation triggers TANK-binding kinase 1 (TBK1) phosphorylation, driving NF-κB nuclear translocation and subsequent release of inflammatory mediators and SASP components. Concurrently, type I interferons induce endothelial pro-inflammatory phenotypes and microvascular dysfunction. Independent of the DNA damage response (DDR) [7], mechanical or metabolic stress activates p38 MAPK via ROS accumulation. Phosphorylated p38 MAPK enhances NF-κB activity through mitogen- and stress-activated protein kinase 1 (MSK1) activation and stabilizes SASP factor mRNAs by suppressing zinc finger protein 36-like 1 (ZFP36L1) [61], extending inflammatory duration. mTORC1 acts as a metabolic hub that inhibits autophagy under oxidative stress, causing the accumulation of damaged mitochondria and persistent mtDNA leakage [62]. It simultaneously enhances SASP factor translation via phosphorylation of eukaryotic translation initiation factor 4E-binding protein 1 (4EBP1) and amplifies NF-κB signaling synergistically with TBK1 [63,64]. Collectively, these pathways drive myocardial fibrosis, microvascular inflammation, and diastolic dysfunction. Thus, NF-κB acts as the master switch initiating SASP, p38 MAPK governs post-transcriptional regulation prolonging inflammation, and mTORC1 perpetuates a metabolic-mitochondrial-inflammatory vicious cycle, cooperatively accelerating HFpEF pathology (Figure 1).

This schematic depicts key signaling cascades linking cellular stress, growth factors and inflammation to cellular senescence and SASP. Growth factor and insulin signaling converge on TSC1/2 to regulate mTORC1; empagliflozin and rapamycin target this axis. Cellular stress activates the p38 MAPK pathway to boost NF-κB and SASP expression. The cGAS-STING pathway triggered by damaged DNA further amplifies inflammatory signaling, while metformin modulates this inflammatory cascade. Senolytics (D+Q, navitoclax) interfere with stress-related signaling. Ultimately, sustained SASP production induces microvascular dysfunction, myocardial fibrosis and diastolic dysfunction, the three major pathological manifestations of HFpEF. Created with BioRender.com.

### 3.3. From Cell Cycle Arrest to Inflammaging: Checkpoint Activation in HFpEF

The p53-p21 and p16-retinoblastoma protein (Rb) pathways, both critically involved in cell cycle regulation, can promote pro-inflammatory phenotypes by modulating SASP secretion [65]. Under chronic inflammation or telomere damage in endothelial cells and cardiac fibroblasts, the DDR activates ataxia telangiectasia mutated (ATM) kinase, which phosphorylates and activates p53, subsequently upregulating p21 transcription. p21 induces cell cycle arrest by inhibiting the CDK2/cyclin E complex, leading to hypophosphorylated Rb protein and repression of E2F-mediated transcription of cell cycle genes [66]. Notably, p53 deficiency counterintuitively promotes the expression of pro-inflammatory SASP components, such as IL-1α/IL-1β, by removing its suppression of NF-κB [67]. During sustained stress, p16, a key senescence maintenance marker, accumulates and inhibits the CDK4/6-cyclin D complex. This maintains Rb in its unphosphorylated state, enabling the formation of a transcriptional repressor complex with E2F that permanently arrests cells in the G0/G1 phase [68]. This cascade drives pre-heart failure pathological manifestations, including endothelial senescence and fibroblast activation [69].

Consequently, therapeutic strategies targeting ATM/p53 or eliminating p16-expressing senescent cells show potential for intervening in SASP and delaying HFpEF progression.

## 4. Central Role of the SASP in HFpEF Pathogenesis

### 4.1. Promoting Cardiac Fibrosis and Increased Stiffness

Cardiac aging involves progressive impairment in physiological and biochemical functions, leading to myocardial fibrosis, increased stiffness, and consequential ventricular diastolic dysfunction, particularly prominent in patients with HFpEF [7]. The SASP plays a pivotal role in this process. SASP promotes myocardial fibrosis and cardiac stiffening primarily through its effects on cardiac fibroblasts, the principal effector cells responsible for extracellular matrix deposition in the heart. The key mechanisms include TGF-β/Smad pathway activation, upregulation of collagen cross-linking enzymes, and disrupted MMP/tissue inhibitor of metalloproteinases (TIMP) balance. TGF-β1 serves as the central fibrogenic mediator, with elevated expression in HFpEF patients during cardiac injury or stress. TGF-β1, secreted by senescent cells, activates TβRII/I receptors on cardiac fibroblasts, triggering Smad2/3 phosphorylation within these cells [70,71]. Activated Smad complexes translocate to the nucleus, initiating pro-fibrotic transcription that upregulates collagen genes (e.g., COL1A1, COL3A1) and lysyl oxidase (LOX) [72]. In activated cardiac fibroblasts, increased LOX expression catalyzes collagen cross-linking, significantly enhancing myocardial stiffness while reducing compliance. Clinical evidence indicates LOX levels rise with age and correlate positively with myocardial fibrosis severity in HFpEF myocardium [31].

Furthermore, SASP disrupts extracellular matrix homeostasis by altering the MMP/TIMP equilibrium. TGF-β1 signaling suppresses MMPs while enhancing TIMP1 expression, reducing collagen degradation, and promoting abnormal accumulation [73,74]. This pathway concurrently induces alpha-smooth muscle actin (α-SMA) expression, driving fibroblast-to-myofibroblast transdifferentiation [75]. SASP-derived inflammatory factors like TNFα additionally activate latent TGF-β1 through integrin αvβ6 [76], establishing a fibrosis-amplifying feedback loop. Sustained TGF-β signaling accelerates cellular senescence by suppressing telomerase reverse transcriptase (TERT) and further exacerbates cardiac dysfunction and structural remodeling via senescence-secreted SASP factors [77]. In HFpEF patients, enhanced LOX activity and MMP/TIMP imbalance critically contribute to adverse structural alterations and functional decline. Consequently, LOX inhibition and MMP/TIMP rebalancing represent promising therapeutic strategies for ameliorating myocardial fibrosis and improving cardiac function in HFpEF.

Collectively, SASP drives myocardial fibrogenesis and stiffness through these interconnected pathways, establishing its central role in HFpEF pathogenesis. Targeting these mechanisms constitutes a promising research avenue for improving HFpEF outcomes.

### 4.2. Microvascular Dysfunction and Rarefaction

Microvascular dysfunction plays a critical role in the development and progression of HFpEF, with endothelial cells lining the coronary microvessels serving as the primary site of injury. This impairment typically manifests as disruption of endothelial tight junctions, reduced NO bioavailability, and excessive secretion of angiogenesis inhibitors, notably TGF-β and thrombospondin-1 (TSP-1) [44]. In coronary microvascular endothelial cells, tight junctions are essential for maintaining microvascular permeability and stability. In HFpEF patients, endothelial cell dysfunction commonly features compromised tight junctions [78]. This deterioration is associated with a chronic low-grade inflammatory state, where cytokines like TNF-α and IL-6 activate endothelial cells, prompting further release of inflammatory mediators that exacerbate microvascular damage [44]. Microvascular rarefaction is frequently observed in HFpEF, correlating with myocardial hypoperfusion and cardiac remodeling, highlighting endothelial protection and repair as potential therapeutic targets.

In endothelial cells, reduced NO bioavailability—a key vasodilator produced by these cells—directly contributes to microvascular dysfunction [79]. Oxidative stress (O_2_^−^ quenching NO), inflammation (TNF-α phosphorylating inhibitory eNOS sites) [80,81], and cellular aging (SASP suppressing endothelial nitric oxide synthase transcription) collectively accelerate NO degradation while impairing its synthesis. This inhibits the soluble guanylate cyclase-cyclic guanosine monophosphate (sGC-cGMP) pathway [82], oxidizing sGC and reducing protein kinase G (PKG) activity, ultimately increasing myocardial stiffness and impairing diastolic function. Enhancing NO bioavailability through pharmacological intervention may therefore ameliorate HFpEF pathophysiology. Excessive secretion of angiogenesis inhibitors like TSP-1 critically drives microvascular rarefaction in HFpEF [35]. TSP-1 inhibits endothelial cell proliferation and migration while promoting apoptosis and inflammation [83]. In endothelial cells, TSP-1 binding to CD47 receptors suppresses SIRT1 deacetylase activity, enhancing p53 acetylation and promoting endothelial cell senescence [84,85]. This process amplifies through SASP, forming a detrimental feedback loop closely linked to cardiac functional decline and poor prognosis [86]. Targeting TSP-1 may concurrently reduce senescent cell burden and improve vascular dysfunction, offering a novel therapeutic strategy.

Collectively, microvascular dysfunction and rarefaction significantly contribute to HFpEF pathogenesis through interacting mechanisms, disrupted endothelial tight junctions, diminished NO bioavailability, and excessive angiogenesis inhibitor secretion, culminating in inadequate cardiac perfusion and functional deterioration. Future research should prioritize deeper mechanistic understanding and targeted interventions to improve HFpEF clinical outcomes.

### 4.3. Impaired Cardiomyocyte Function

Functional impairment of cardiomyocytes critically contributes to aging and heart failure pathogenesis, primarily through three interconnected mechanisms: disrupted Ca^2+^ handling, mitochondrial dysfunction, and reduced myofilament Ca^2+^ sensitivity.

#### 4.3.1. Dysregulation of Calcium Homeostasis

Disrupted intracellular Ca^2+^ handling initiates a cascade of dysfunction. During systole, Ca^2+^ leakage occurs through hyperphosphorylated or oxidatively damaged ryanodine receptor 2 (RyR2). This leakage is exacerbated by diminished SR Ca^2+^ stores, resulting from Sarcoplasmic/Endoplasmic Reticulum Calcium ATPase 2a (SERCA2a) dysfunction, which collectively reduces Ca^2+^ transient amplitude [87,88]. During diastole, impaired SERCA2a activity, resulting from downregulated expression and phospholamban (PLB) disinhibition, delays Ca^2+^ reuptake, prolonging ventricular relaxation [89]. Sustained cytosolic Ca^2+^ overload activates calpain-mediated myofilament degradation and, synergistically with ROS nitric oxide (NO), induces mitochondrial permeability transition pore (mPTP) opening, impairing ATP synthesis [90]. Furthermore, Ca^2+^ overload activates the Ca^2+^/calmodulin-dependent protein kinase II δ (CaMKIIδ) and calcineurin–nuclear factor of activated T cells (NFAT) pathways, driving pathological cardiomyocyte hypertrophy and promoting SASP secretion that indirectly accelerates myocardial fibrosis [91,92].

#### 4.3.2. Mitochondrial Dysfunction and the SASP Amplification Loop

Mitochondrial dysfunction, a hallmark of aging cardiomyocytes, involves reduced activity of electron transport chain (ETC) complexes I/III, which decreases ATP production while increasing ROS generation and mitochondrial DNA (mtDNA) leakage [93,94]. In cardiomyocytes and endothelial cells, leaked mtDNA activates the cGAS-STING pathway, triggering the release of SASP factors such as IL-6 and TNF-α, thereby establishing a “mitochondrial damage-SASP amplification” axis [95]. SASP components, in turn, exacerbate mitochondrial collapse via multiple mechanisms: IL-6 suppresses peroxisome proliferator-activated receptor gamma coactivator 1-α (PGC-1α) synthesis, impairing mitochondrial biogenesis; TNF-α induces NADPH oxidase 4 (NOX4) overexpression, depleting antioxidant defenses; and TGF-β promotes excessive fission through dynamin-related protein 1 (DRP1) phosphorylation. The resulting ATP deficiency further inhibits SERCA2a, aggravating diastolic dysfunction, while persistent mtDNA leakage activates Toll-like receptor 9 (TLR9) and NF-κB, perpetuating SASP release [96,97].

#### 4.3.3. Reduced Myofilament Ca^2+^ Sensitivity

The contractile response of cardiomyocytes is also compromised by a decline in myofilament Ca^2+^ sensitivity, a key determinant of contractile force in heart failure and aging [98]. This reduction is driven by altered phosphorylation of the troponin complex and changes in regulatory protein expression [44]. Pro-inflammatory mediators derived from the SASP likely directly impair myofilament function, accelerating this loss of sensitivity. Therapeutic strategies aimed at restoring myofilament Ca^2+^ sensitivity therefore represent a promising avenue for improving cardiac output in heart failure.

## 5. Therapeutic and Translational Potential

### 5.1. Therapeutic Strategies of Senolytics and Senomorphics

Senolytics represent a class of targeted therapeutics that selectively clear senescent cells (SCs) by disrupting their pro-survival pathways [99,100], such as B-cell lymphoma 2/B-cell lymphoma-extra-large (BCL-2/BCL-xL) and p53-p21, to induce apoptosis [101]. This reduction in SC burden alleviates SASP-mediated tissue damage. Senomorphics comprise agents that inhibit SASP secretion without eliminating SCs, primarily aiming to block SASP-driven inflammation, fibrosis, and metabolic dysregulation to delay tissue dysfunction [102]. Both strategies demonstrate therapeutic potential for mitigating age-related cardiac pathologies through SC clearance or SASP suppression. However, enthusiasm for senotherapeutics must be tempered by their potential risks and unresolved challenges. A primary concern is the lack of cell-type specificity: current senolytics may inadvertently eliminate essential non-senescent cells that share similar survival pathways, potentially impairing tissue homeostasis and processes such as wound healing. Furthermore, by clearing senescent cells, which can play context-dependent roles in immune surveillance and tumor suppression, there is a theoretical risk of promoting malignancy or increasing susceptibility to infections. Senomorphic agents, while avoiding direct cell killing, carry the risk of suppressing beneficial, transient SASP responses necessary for tissue repair or immune coordination. Other key hurdles include defining optimal treatment regimens (intermittent vs. continuous), identifying robust biomarkers for patient selection and treatment monitoring, and understanding the long-term consequences of modulating senescence in a chronically ill, aging HFpEF population. Specific senolytics, including the dasatinib-quercetin (D+Q) combination, effectively reduce SC load, attenuate inflammation, and improve cardiac function by decreasing SASP components in myocardial tissue in preclinical models [103,104].

Similarly, the FOXO4-interfering peptide (FOXO4-DRI) exerts cardioprotection through SC clearance and SASP reduction [105]. Despite extensive mechanistic studies on these agents in cellular pathology, research remains notably limited in HFpEF, a condition characterized by ventricular remodeling and chronic cardiac dysfunction. Table 2 summarizes the effects of common senolytics and senescent cell modulators on the heart.

However, translating these promising preclinical findings into clinical benefit requires evidence from human trials. Pioneering early-phase studies have begun to bridge this gap. For instance, the senolytic combination dasatinib and quercetin (D+Q) has demonstrated feasibility and reduced circulating SASP factors in patients with diabetic kidney disease [106]. Although direct evidence in HFpEF populations is awaited, this provides a crucial proof-of-concept. Furthermore, drugs with potential senomorphic properties, such as SGLT2 inhibitors, have shown definitive cardiovascular benefit in large-scale HFpEF outcome trials (e.g., EMPEROR-Preserved) [107,108], with mechanisms that may partly involve attenuating SASP-related inflammation. Collectively, SC-targeted therapies offer novel treatment avenues for age-related diseases, including heart failure, and represent a promising frontier in clinical research.

**Table 2:** Effects of senolytics and senescent cell modulators on the heart.

Drug(s)	Mechanism of Action	Cardiac Impact	Refs.
Senolytics			
Dasatinib (D) + Quercetin (Q)	Dasatinib inhibits tyrosine kinases, while quercetin activates the mitochondrial apoptotic pathway and inhibits the PI3K pathway	Improves ventricular diastolic dysfunction	[109]
Navitoclax (ABT-263)	Inhibits anti-apoptotic proteins and reduces SASP factors	Reduces senescent cardiomyocytes and improves ventricular diastolic function in aged mice, preventing adverse cardiac remodeling	[110,111]
Fisetin	Targets the BCL-2 family, PI3K, and other molecules; induces apoptosis of various senescent cells; reduces the secretion of SASP factors	Inhibits phenylephrine (PE)-induced cardiomyocyte hypertrophy; reduces oxidative stress	[112]
Nintedanib	Inhibits the Janus kinase 2/signal transducer and activator of transcription 3 (JAK2/STAT3) pathway; reduces SASP-related inflammatory responses by clearing senescent fibroblasts	Reduces myocardial and systemic inflammation by inhibiting pro-inflammatory subsets and promoting regulatory T cells (Treg)	[113]
Heat Shock Protein 90 (HSP90) Inhibitors	17-Dimethylaminoethylamino-17-demethoxygeldanamycin (17-DMAG) disrupts the HSP90-protein kinase B (AKT) interaction	Attenuates cardiac hypertrophy and improves cardiac function	[114,115]
Curcumin Analog	A naturally occurring p300-histone acetyltransferase (p300-HAT) inhibitor; inhibits p300-HAT activity, degrades BCL-xL and myeloid cell leukemia 1 (Mcl-1), suppresses SASP secretion	Significantly inhibits the development of heart failure in mice	[116]
Senomorphics			
Rapamycin	Inhibits the mammalian target of mTORC1 pathway and regulates T-cell function	Improves cardiac function and inhibits cardiac remodeling	[117,118]
Ruxolitinib	Inhibits the JAK1/2-STAT3 pathway	Improves mitochondrial dysfunction, oxidative stress, and ATP levels in mouse cardiac tissue	[119]
Metformin	Inhibits the NF-κB pathway	Improves myocardial oxygen consumption and reduces key markers of heart failure	[120]
p38MAPK Inhibitors (BIRB796, MW150)	Inhibits the activation of p38MAPK	Attenuates cardiac inflammation and cardiac dysfunction in mice with HFpEF; delays cardiomyocyte hypertrophy and fibrosis, and improves cardiac function	[121,122]
Resveratrol	Indirectly inhibits (relevant pathways) by activating nuclear factor erythroid 2-related factor 2 (Nrf2); activates the Sirt1/p53 pathway in heart failure	Improves cardiac function	[47,123]
Melatonin	Interferes with the poly (ADP-ribose) polymerase 1 (PARP1)-CREB-binding protein (CBP) interaction	Improves symptoms of heart failure	[124,125,126,127]
Ubiquitin-Specific Protease 7 (USP7) Inhibitors	USP7 directly binds to mothers against decapentaplegic homolog 3 (SMAD3) via its ubiquitin-like (UBL) domain and cysteine at position 223 of USP7, promoting endothelial-mesenchymal transition (EndMT) and cardiac fibrosis. USP7 inhibitors directly suppress SASP factors and reduce the expression of IL-6 and other molecules	Endothelium-specific USP7 knockout improves the HFpEF phenotype and reduces cardiac fibrosis	[128]
Proteolysis-Targeting Chimeras (PROTAC)	Reduces the expression of SASP factors such as IL-6	Attenuates cardiomyocyte lesions, mitochondrial dysfunction, and apoptosis	[129]

### 5.2. Targeted Inhibition of Specific SASP Factors

IL-1β is a key pro-inflammatory cytokine within the SASP, whose overexpression impairs myocardial contraction/relaxation and promotes maladaptive cardiac remodeling. In heart failure, IL-1β release closely correlates with cardiac remodeling and functional decline. Neutralizing antibodies targeting IL-1β, such as canakinumab, have shown anti-inflammatory and cardiovascular protective effects in a post-myocardial infarction population with residual inflammatory risk (elevated hsCRP) [130]. Although not yet evaluated in dedicated HFpEF trials, this evidence provides a strong rationale for investigating IL-1β inhibition as a potential strategy to modulate the SASP and improve outcomes in HFpEF, a syndrome profoundly driven by chronic inflammation. Beyond attenuating SASP-associated inflammation, IL-1β inhibition may also ameliorate cardiac metabolic dysfunction, offering a novel treatment avenue for age-related heart failure.

The TGF-β signaling pathway plays a central role in cardiac remodeling and fibrosis during heart failure progression [131,132]. SASP components markedly elevate TGF-β expression, accelerating cardiac fibrosis and functional impairment. Inhibitors of TGF-β signaling, such as small-molecule kinase inhibitors, represent potential therapeutics that attenuate fibrosis, mitigate remodeling, and improve cardiac function by disrupting TGF-β-mediated signal transduction [133]. These inhibitors suppress activation and proliferation of cardiac fibroblasts, reduce cardiomyocyte apoptosis, and decrease SASP factor production, constituting a multi-faceted strategy against heart failure. TGF-β pathway inhibition may further ameliorate metabolic dysfunction and delay age-related heart failure progression.

Chemokines significantly contribute to SASP by promoting immune cell infiltration and activation, exacerbating cardiac inflammation [134]. Chemokine receptor antagonists effectively inhibit the recruitment of inflammatory cells, reduce cardiac injury, and diminish fibrosis [135]. These agents improve cardiac function and suppress SASP factor release, revealing a targeted strategy for modifying pathological states in age-related heart failure. Experimental studies suggest that chemokine receptor antagonism may also indirectly reduce oxidative stress and improve metabolic function, highlighting its broad therapeutic potential for heart failure treatment.

### 5.3. Integration with Conventional Therapies

Heart failure management centers on lifestyle interventions and pharmacotherapy [136]. Exercise training, particularly high-intensity interval training (HIIT), significantly improves cardiac function and exercise capacity in HFpEF patients while reducing senescence markers, thereby enhancing cardiovascular health. This metabolic optimization proves especially beneficial for HFpEF patients with obesity or metabolic syndrome, positioning exercise as both a cardioprotective strategy and countermeasure against age-related pathology.

Caloric restriction (CR), validated across multiple models for its anti-aging effects, alleviates cardiac burden and improves systemic metabolism in HFpEF [11,137]. Mechanistically, CR activates adenosine monophosphate-activated protein kinase (AMPK) while inhibiting the mechanistic target of the mTOR pathway, boosting autophagic clearance of senescent cells and reducing inflammatory burden [138]. Pharmacologically, RAAS inhibitors constitute standard therapy for HFpEF by lowering blood pressure, reducing cardiac load, and suppressing myocardial fibrosis [139].

SGLT2 inhibitors significantly decrease hospitalization and mortality by enhancing ketone utilization, reducing sodium reabsorption, and improving myocardial energetics [140]. Emerging nanotechnology enables precision delivery of therapeutics to senescent cells via engineered nanoparticles functionalized with targeting ligands, maximizing drug bioavailability while minimizing off-target effects [141].

These systems allow controlled release kinetics for dynamically modulating senescent cell burden. Key SASP factors, including IL-1α/IL-1β, IL-6, and TNF-α, show elevated expression in heart failure, where they accelerate cardiomyocyte dysfunction and disease progression through pro-inflammatory cascades [85]. Integrating foundational interventions (exercise/CR), standard pharmacotherapies (RAAS/SGLT2 inhibitors), and advanced approaches (SASP-directed nanotherapy) creates a multi-target framework poised to enhance HFpEF clinical outcomes synergistically.

### 5.4. SASP Biomarkers: Current Status and Clinical Potential

Despite progress in therapeutic development, a major barrier to translating senotherapies into clinical practice for HFpEF is the lack of validated biomarkers for patient stratification and treatment monitoring. Promising candidates are emerging from the SASP itself. Growth differentiation factor 15 (GDF-15), a stress-responsive cytokine elevated in senescent cells and chronic inflammation, is strongly associated with HFpEF diagnosis, disease severity, and adverse outcomes [142]. GDF-15, along with senescent cell-derived EV cargoes, represents a viable candidate for monitoring senescent cell burden and therapeutic response [143,144]. These EVs carry SASP factors (e.g., TNF-α) and non-coding RNAs and can serve as a “liquid biopsy” to assess senescent cell burden in tissues [145]. Other SASP components such as TGF-β and MMP-2/9 have been investigated but lack specificity for senescence versus general inflammation. Future efforts should validate these biomarkers in longitudinal HFpEF cohorts to guide personalized therapy.

## 6. Discussion

HFpEF represents a complex and increasingly prevalent cardiac condition with incompletely elucidated pathophysiology [136]. Advancing our understanding and treatment of this syndrome requires addressing several interconnected translational challenges.

Current HFpEF research primarily relies on animal models that exhibit significant limitations [146]. They frequently fail to recapitulate key human disease features, including the full spectrum of metabolic comorbidities (e.g., obesity, diabetes), nutritional states, and their integrated impact on cardiac function. More critically, HFpEF is a disease closely associated with aging, in which SASP mediates multi-organ dysfunction through the secretion of pro-inflammatory factors and chemokines. However, due to differences in aging responses and pathway activation across species, animal models often fail to accurately mimic the dynamic changes in human SASP [4,5]. This fundamental disconnect between preclinical models and human pathophysiology constitutes a major barrier to the identification of robust therapeutic targets and the prediction of clinical efficacy.

To overcome the limitations of model systems and capture the human disease state, emerging technologies are essential. Single-cell RNA sequencing enables the resolution of senescence and SASP heterogeneity across distinct cardiac cell types (e.g., cardiomyocytes, fibroblasts, endothelial cells, immune cells), revealing unique signatures that collectively drive pathology.

**Figure 2:**
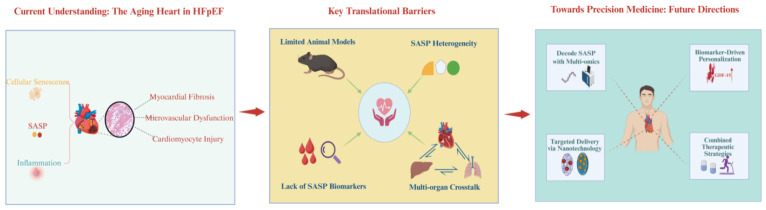
Translational Challenges and Future Directions in Senescence-Targeted HFpEF Therapy.

The recognition of HFpEF as a disease of multi-organ aging presents unique challenges and opportunities for clinical translation. Elderly patients, who constitute the majority of the HFpEF population, exhibit distinct clinical manifestations, comorbid profiles, and potential differential responses to therapies. This heterogeneity necessitates the design of age-optimized clinical trials that incorporate comprehensive geriatric assessments. In the future, combining exosome-based diagnostics, interventions targeting specific SASP pathways, and targeted regulation of multi-organ aging networks holds promise for pioneering new precision treatment approaches for this complex disease (Figure 2).

This schematic primarily describes how cellular senescence, inflammation, and the SASP drive key pathological features of HFpEF, including myocardial fibrosis, microvascular dysfunction, and cardiomyocyte injury. Currently, the main translational barriers to effective senotherapies are limited animal models, SASP heterogeneity, a lack of validated SASP biomarkers, and multi-organ crosstalk. Corresponding future strategies to overcome these hurdles include decoding SASP complexity via multi-omics profiling, targeted delivery based on nanotechnology, biomarker-driven personalized treatment, and combined therapeutic regimens. Created with BioRender.com.

In addition, the clinical translation of senotherapeutics faces several conceptual and practical limitations. A primary concern is the lack of cell-type specificity inherent to current senolytics. These agents target pro-survival pathways that are upregulated in, but not exclusive to, senescent cells. Consequently, they risk eliminating essential, non-senescent cells that share similar dependencies, potentially impairing tissue homeostasis, regeneration, and wound healing processes. Furthermore, the biological role of senescent cells is context-dependent; they are involved in physiological processes such as wound healing, embryonic development, and, crucially, tumor suppression and immune surveillance. Indiscriminate elimination of senescent cells may therefore carry a theoretical risk of compromising antitumor immunity or increasing susceptibility to certain infections [102,147].

Senomorphic strategies, while avoiding direct cell killing, present their own set of challenges. By broadly suppressing the SASP, these agents risk inhibiting beneficial, transient secretory responses that are necessary for coordinated tissue repair, immune cell recruitment, and pathogen clearance. This underscores the need for more refined agents that can discriminate between pathological, chronic SASP and beneficial, acute secretory signals. Beyond these biological considerations, significant translational hurdles remain. Defining optimal treatment regimens (e.g., intermittent dosing to allow recovery versus chronic administration) is unresolved. The field also critically lacks validated, clinically accessible biomarkers to reliably identify patients with high senescent cell burden (“senescence load”) and to monitor the efficacy of senolytic or senomorphic interventions. Finally, the long-term safety and consequences of modulating senescence, particularly in a frail, elderly, and multimorbid HFpEF population, are entirely unknown and warrant careful evaluation in future trials [28,102].

Limitations of this review should be acknowledged. The conclusions presented in this review are predominantly based on preclinical and mechanistic studies derived from animal models, which may not fully recapitulate the complexity and heterogeneity of human HFpEF. Furthermore, this narrative review, while comprehensive, did not employ a systematic literature search methodology, which may introduce selection bias. The interpretation of therapeutic potential, particularly for senolytics and senomorphics, should therefore be tempered by these constraints and awaits validation in well-designed clinical trials.

## 7. Conclusions

The pathogenesis of HFpEF is fundamentally linked to cellular senescence and the SASP, where mediators, including IL-6, TNF-α, TGF-β, and exosome-carried factors, orchestrate myocardial fibrosis, microvascular dysfunction, and cardiomyocyte injury. Preclinical studies demonstrate that senolytics and senomorphics attenuate HFpEF by reducing SASP burden. However, clinical translation requires overcoming three barriers: limited cell-type specificity of current senotherapeutics, absence of validated SASP biomarkers for therapeutic monitoring, and insufficient adaptation to geriatric complexities in HFpEF populations.

Future efforts should prioritize the multi-omics decoding of SASP heterogeneity, the development of targeted delivery systems, and age-optimized trial designs that integrate comprehensive geriatric assessment. Addressing these challenges will bridge mechanistic insights into effective clinical strategies for this growing patient population.

## Figures and Tables

**Table 1:** Protein components of SASP and their pathological roles in HFpEF.

Category	Key Mediators	Primary Functions	HFpEF-Specific Effects	Refs.
Interleukins/Cytokines	IL-6, IL-1β, TNF-α	Activate JAK-STAT/NF-κB pathways; Induce systemic inflammation	Drives myocardial inflammation, insulin resistance; Promotes sarcopenia and adipose dysfunction	[5,45]
Chemokines	IL-8 (CXCL8) MCP-1 (CCL2)	Recruit neutrophils/monocytes via CXCR2/CCR2 receptors	Causes immune cell infiltration in cardiac microvasculature; Amplifies endothelial dysfunction	[46]
Growth Factors	TGF-β, VEGF	TGF-β: Fibrosis activation via Smad3; VEGF: Angiogenesis regulation;	TGF-β: Core driver of myocardial stiffness; VEGF: Induces disorganized angiogenesis	[47]
Matrix Proteases	MMP-2, MMP-9, PAI-1	Degrades collagen/fibronectin; inhibits fibrinolysis	MMPs: Disrupt ECM homeostasis, diastolic impairment; PAI-1: Promotes fibrin deposition and microvascular thrombosis	[48,49]

## Data Availability

Not applicable.
